# A Combination Therapy in a Rare Case of Adult-Onset Autoimmune Enteropathy

**DOI:** 10.7759/cureus.42538

**Published:** 2023-07-27

**Authors:** Bushra Amer, Waleed H Khozaig, Monia T Alhadad, Nadir Abdelrahman

**Affiliations:** 1 Department of Family Medicine, Michigan State University College of Human Medicine, East Lansing, USA; 2 Department of Gastroenterology, Prince Mohammed Bin Abdulaziz Hospital, Riyadh, SAU; 3 Department of Family Medicine-Geriatrics, Michigan State University College of Human Medicine, East Lansing, USA

**Keywords:** villous atrophy, azathioprine, methylprednisolone, intractable diarrhea, autoimmune enteropathy

## Abstract

Autoimmune enteropathy (AIE) is a differential diagnosis of incurable chronic diarrhea, malnutrition, and weight loss. This type of diarrhea is associated with protein enteropathy that usually affects the small intestine. The diagnosis of AIE is based on chronic diarrhea, malabsorption, specific histological result, antibodies against enterocytes, and excluding similar conditions. In this case, a 28-year-old female presented with diarrhea, lower limb edema, weight loss, and electrolyte imbalances. Endoscopic examination demonstrated duodenal villous atrophy, while duodenal biopsies revealed villous blunting, scattered intraepithelial lymphocytes, and crypt hyperplasia in the lamina propria. The patient was treated with immunosuppressive treatment including methylprednisolone and azathioprine, achieving clinical remission.

## Introduction

The diagnostic criteria for autoimmune enteropathy (AIE) include chronic diarrhea associated with circulating gut autoantibodies and varying levels of villous atrophy of the small intestine [1]. The symptoms are not specific and can occur in conjunction with other autoimmune diseases [2]. In adults, the diagnostic criteria for AIE include chronic diarrhea, malabsorption, specific histological findings of complete villous blunting in the small bowel, and the elimination of all other causes of villous atrophy. However, the absence of antibodies to enterocytes and goblet cells does not exclude a diagnosis of AIE, although their presence is significant [3]. Patients usually warrant immunosuppressive drugs, and some of them require total parenteral nutrition since they do not respond to dietary modification, including a gluten-free diet [4]. The term AIE was proposed by Unsworth and Walker-Smith in 1985 to describe a condition of prolonged diarrhea with the presence of autoantibodies against the intestinal epithelium in combination with pathological processes in other organs, in addition to the presence of serological markers compatible with autoimmunity, in infants [5]. In 1997, Corazza and colleagues reported autoimmune enteropathy in adults for the first time. Two adult individuals were identified with antibodies against enterocytes, which were suspected to be gluten-resistant enteric disease. A new feature in gastroenterology was introduced as a result of the description of the feature and potential autoimmune cause of this disorder [6]. The diagnosis of AIE is challenging due to considerable overlap with other diseases characterized by villous atrophy and medication-associated enteropathy [7-9]. In this report, we present a case of a 28-year-old female who presented with symptoms of diarrhea, lower limb edema, weight loss, and electrolyte abnormalities. The patient showed a positive response to combination therapy with methylprednisolone and azathioprine.

## Case presentation

**Figure 1:**
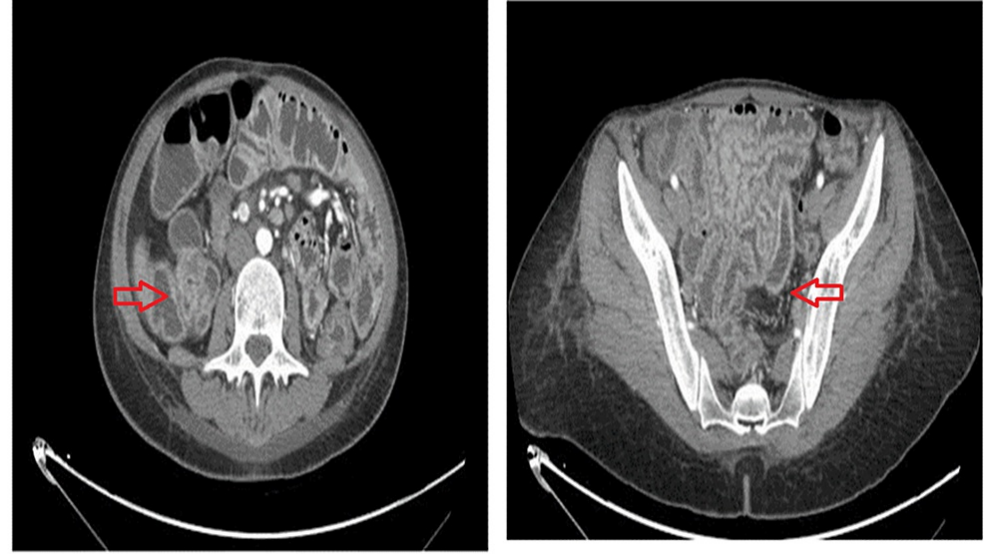
CT showing the increased enhancement of gastric, jejunal, and colonic mucosa, which demonstrated an inflammatory process, particularly in the jejunum and to lesser extent in the terminal ileum. CT: computed tomography

A 28-year-old female presented to the gastroenterology department with bilateral lower limb swelling and non-bloody diarrhea accompanied by steatorrhea that had been persisting for a month. Furthermore, she had an unintentional 10 kg weight loss (15% of body weight). Her physical examination was normal, except for pitting edema up to both thighs besides nail clubbing. She has a history of a complicated pregnancy with repeated UTI, for which early delivery at eight months was required. Her family history is unremarkable. Laboratory investigations revealed findings of iron deficiency anemia, hypokalemia, and folate deficiency. Anti-tissue transglutaminase antibody (anti-tTG Ab) showed a slight elevation, while the serology results for HIV, hepatitis B surface antigen (HBsAg), hepatitis C virus antibody (HCV Ab), and stool cultures were negative. Abdominal ultrasound showed mild ascites and hepatosplenomegaly, while the computed tomography (CT) scan revealed increased enhancement of gastric, jejunal, and colonic mucosa, which demonstrated an inflammatory process, particularly in the jejunum and to a lesser extent in the terminal ileum (Figure 1).

**Figure 2:**
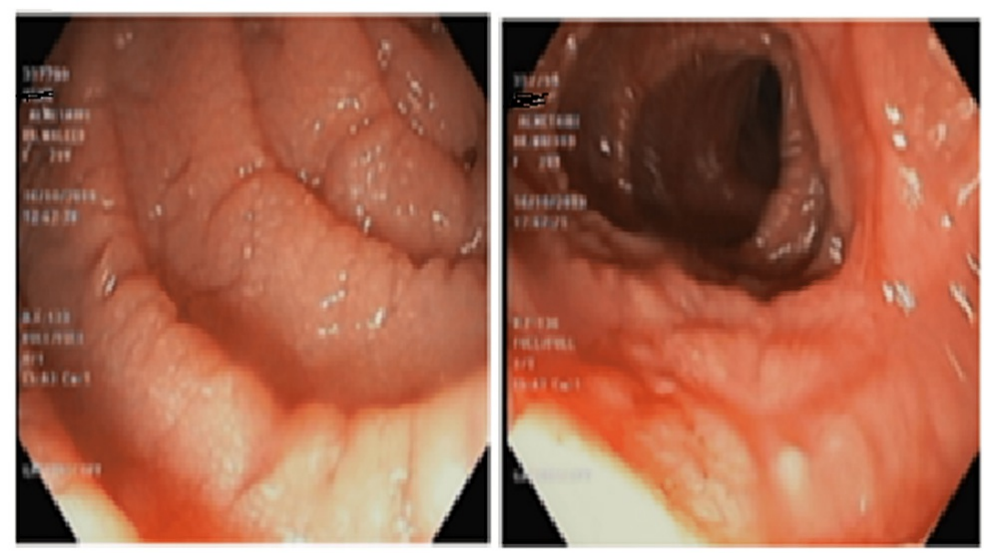
Esophagogastroduodenoscopy showing duodenal villous atrophy beside focal erythema.

Next, the esophagogastroduodenoscopy showed duodenal villous atrophy with focal erythema (Figure 2).

**Figure 3:**
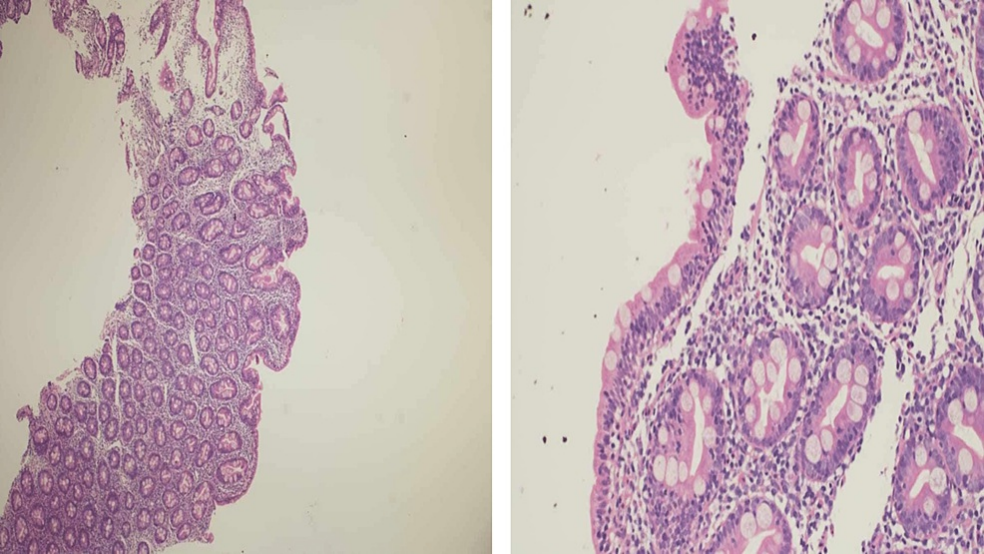
The histopathological evaluation showing villous blunting, scattered intraepithelial lymphocytes, crypt hyperplasia in the lamina propria, and significant lymphoplasmacytic infiltration admixed with neutrophils, as well as eosinophils.

Villous blunting was observed in the histopathological evaluation of duodenal biopsies (Figure 3), with scattered intraepithelial lymphocytes (37/100 enterocytes), crypt hyperplasia in the lamina propria, and significant lymphoplasmacytic infiltration admixed with neutrophils, as well as eosinophils (Marsh 3b).

Despite being placed on a gluten-free diet and receiving alendronate for osteoporosis, the patient did not experience any clinical improvement. The patient's treatment was initiated with methylprednisolone, leading to significant improvement in symptoms and a slight increase in weight. However, after one month, the patient required readmission due to the recurrence of diarrhea and hypokalemia. Subsequently, the patient started taking a combination of methylprednisolone and azathioprine and experienced rapid clinical improvement.

## Discussion

AIE is a rare condition characterized by immune-mediated injury, resulting in intractable diarrhea and small intestine mucosal atrophy. Despite dietary modifications, including a gluten-free diet, patients remain unresponsive to treatment [2,4]. Although primarily seen in pediatric patients, AIE is also recognized to occur in adults [10]. Furthermore, the disease frequently occurs in combination with other autoimmune conditions [11]. Diagnosing AIE is challenging due to the nonspecific nature of its symptoms [2,11]. Chronic diarrhea, malabsorption, specific small bowel histology findings, the exclusion of other causes of villous atrophy, and the detection of antibodies against enterocytes are the criteria for the diagnosis [4]. Villous blunting, deep crypt lymphocytosis, increased crypt apoptotic bodies, and minimal intraepithelial lymphocytosis were observed in small bowel histology [4]. Capsule endoscopy is a useful tool for assessing diffuse inflammation in the small bowel and identifying mucosal scalloping, fissuring, and mosaic patterns [4,12]. We report a case of chronic diarrhea and significant weight loss in a patient with adult-onset AIE, whose esophagogastroduodenoscopy revealed duodenal villous atrophy with focal erythema and slightly elevated antibodies against enterocytes. It is crucial to exclude other reasons for small bowel villous atrophy to confirm the diagnosis. AIE is caused by the disruption of gut humoral and cellular immune function with an underlying defect in the regulatory T-cell system [2]. Initiating early diagnosis and starting the appropriate immunosuppressive therapy and nutritional support are crucial for managing the disease effectively, as it is the gold standard [11,13]. A clinical improvement was reported in 60% of patients following eight weeks of immunosuppressive treatment [2,4]. Other medications include antitumor necrosis factors [4,10,13]. Therapeutic management in our case was challenging and involved methylprednisolone and azathioprine. The response was rapid, complete, and sustained.

## Conclusions

In this case, a 28-year-old female presented with diarrhea, lower limb edema, weight loss, and electrolyte imbalances. Endoscopic examination demonstrated duodenal villous atrophy, while duodenal biopsies revealed villous blunting, scattered intraepithelial lymphocytes, and crypt hyperplasia in the lamina propria. The patient was treated with immunosuppressive treatment including methylprednisolone and azathioprine, achieving clinical remission.
